# Owners’ experiences of caring for cats with chronic kidney disease in the UK

**DOI:** 10.1177/1098612X251314769

**Published:** 2025-04-16

**Authors:** Jenna Elliott, Holly Reyes-Hughes, Angie Hibbert, Emily Blackwell, Natalie C Finch

**Affiliations:** 1 University of Bristol, Bristol, UK; 2 Highcroft Veterinary Group, Bristol, UK; 3 The Feline Centre, Langford Vets, University of Bristol, Bristol, UK

**Keywords:** Kidney, owners, experiences, emotions, management, caregiver burden

## Abstract

**Objectives:**

The aim of the present study was to describe current practices in the management of feline chronic kidney disease (CKD), and the experiences and emotions of caring for a cat with CKD, reported by UK cat owners.

**Methods:**

A survey study was conducted of UK owners of cats with CKD that included multiple-choice, Likert scale and open questions reported using descriptive statistics.

**Results:**

Responses from 100 UK owners of cats with CKD were included in the study. Of the owners, 73% had knowledge of their cat’s International Renal Interest Society stage and 76% were feeding their cat a renal diet, with 70% reporting that veterinary recommendation had influenced this decision. Of the owners, 35% were administering phosphate binders, 26% natural supplements and 25% antiproteinuric therapies to their cat. Of concern was the high number of owners reporting feelings of anxiety (99%) as well as other negative emotions associated with caring for their cat with CKD. Extreme worry, anxiety or concern were high at both the time of CKD diagnosis and survey completion, related to disease prognosis (72% and 50%, respectively) and cats’ quality of life (63% and 46%, respectively). Many owners agreed that their daily routine had changed a lot since their cat was diagnosed with CKD (66%) and that caring for their cat negatively impacted or restricted their own life (37%).

**Conclusions and relevance:**

Although owners’ knowledge and understanding of their cat’s CKD and appropriate management was generally in line with expert guidelines, the finding that many owners reported experiencing feelings of anxiety associated with caring for their cat, suggesting caregiver burden, was concerning. This highlights the impact that veterinary professionals, including veterinary nurses, may have in providing greater education and support to enhance the relationship and communication with owners of cats with CKD, thus helping to alleviate anxiety and ensure ongoing successful CKD management.

## Plain language summary

We report how UK owners of cats with chronic kidney disease (CKD) manage their cat’s CKD and the experiences and emotions they felt caring for their cat with CKD. We distributed a survey to UK owners of cats via social media and the Bristol Cats study. The survey questions included multiple-choice, Likert scale and open questions. The survey was split into five sections: (1) owner demographic information; (2) cat’s age, sex and breed; (3) information related to the cat’s CKD; (4) information related to the management of the cat’s CKD; and (5) owners’ experiences and emotions of caring for a cat with CKD. We included responses from 100 UK owners of cats with CKD in the study. Of the owners, 73% had knowledge of their cat’s CKD stage, 76% were feeding their cat a kidney support diet, 35% were administering supplements to bind phosphate in the gut and 26% were administering natural supplements. Of concern was the high number of owners reporting feelings of anxiety (99%) as well as other negative emotions associated with caring for their cat with CKD. Extreme worry, anxiety or concern were high at both the time of CKD diagnosis and survey completion. The extreme worry, anxiety or concern was related to disease prognosis and cats’ quality of life. Many owners agreed that their daily routine had changed a lot since their cat was diagnosed with CKD (66%) and that caring for their cat negatively impacted or restricted their own life (37%). Although owners’ knowledge and understanding of their cat’s CKD and management for the disease was in line with expert guidelines, the finding that many owners reported experiencing feelings of anxiety associated with caring for their cat, suggesting caregiver burden, was concerning. This highlights the impact that veterinary professionals, including veterinary nurses, may have in providing greater education and support to enhance the relationship and communication with owners of cats with CKD, thus helping to alleviate anxiety and ensure ongoing successful CKD management.

## Introduction

Chronic kidney disease (CKD) has a prevalence of up to 80% in cats aged 15 years and older,^1^ and is the seventh most common diagnosis in feline medicine.^2^ CKD is defined by chronic structural or functional abnormalities of the kidney and is the consequence of different disease pathways. International Renal Interest Society (IRIS) staging is performed after a confirmed diagnosis of CKD to help guide a veterinarian regarding prognosis and treatment recommendations. Therapeutic strategies aim to delay disease progression and prevent advancement to end-stage renal disease. Optimal management of CKD involves targeted individualised treatment plans requiring shared decision-making with owners and relationship-centred care.^2,3^ Previous studies have reported management practices of owners of cats with CKD.^4,5^ However, owners’ experiences and emotions and how these might influence the care of the cat have not been reported.

In humans, caregiver burden can be associated with chronic illness^6^ and describes the response to difficulties encountered when providing care for an individual. It is associated with both the emotional and physical demands of being a caregiver and can impact on patient care.^6^ Increased stress, burden, symptoms of depression or anxiety, and poorer quality of life consistent with caregiver burden have been reported in owners of pets with chronic or terminal disease.^7,8^ Caregiver burden has been reported in owners of ill cats, of which some cats had kidney disease,^9^ and described in a non-peer-reviewed article written by an owner of a cat with CKD.^10^

To the authors’ knowledge, there are no studies reporting owners’ experiences and emotions associated with caring for a cat with CKD. The aims of the present study were to describe current UK owner practices in the management of feline CKD as well as the experiences and emotions of owners of cats with CKD in the UK.

## Materials and methods

### Survey design

The survey consisted of 35 questions: 26 multiple-choice, seven Likert scale and two open questions (see supplementary material). Six of the multiple-choice questions required respondents to answer a ‘Please specify’ free-text question. Of the seven Likert scale questions, six included an optional open free-text subquestion. Four of the multiple-choice questions allowed the participants to select more than one answer. The survey was split into five sections: (1) owner demographics; (2) cat signalment; (3) cat’s CKD; (4) management; and (5) owner’s experiences and emotions of caring for a cat with CKD.

### Owner recruitment

The online survey platform, Bristol Online Surveys, was used to create and host the survey. Participants were recruited using the social media platform Facebook via a short recruitment advert and link to the survey. The recruitment post was shared across a variety of Facebook groups, including multiple support groups for owners of cats with CKD, and other UK-based cat owner groups. Diagnostic criteria to define CKD were not included. The survey was also shared with owners participating in the Bristol Cats study (https://www.bristol.ac.uk/vet-school/research/projects/cats/). Participants were eligible to complete the survey if they currently owned a cat diagnosed with CKD and lived in the UK. Participants could complete the survey for multiple cats. The survey was open between 15 October 2020 and 15 January 2021.

### Data analysis

The data obtained from the survey responses was downloaded from Bristol Online Surveys in an anonymised format. Analysis of the data consisted of descriptive statistics. Data from the open free-text questions were grouped according to subject.

### Ethical approval

The study was reviewed and approved by the Faculty of Health Sciences Research Ethics Committee (FREC) at the University of Bristol (reference number 111303). Participants consented to study inclusion by reading the information page and continuing to take the survey. Participants were able to withdraw from the study, but only up until the point answers were fully submitted as all responses were anonymised from that point.

## Results

A total of 111 responses were collected from the survey. Of them, 11 responses were excluded as the respondent did not meet the inclusion criteria, leaving a total of 100 responses for analysis.

### Owner demographics

**Figure 1:**
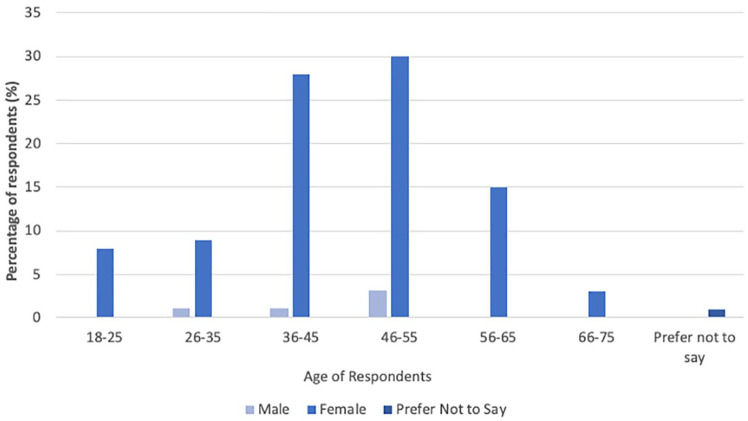
Age and gender of respondents

All included respondents lived in the UK (n = 100, 100%). Of the respondents, 94 (94%) were female, five (5%) were male and one (1%) participant preferred not to say (Figure 1). The age range was 18–75 years (Figure 1). The most common age range was 46–55 years (n = 33, 33%) and the least common was 66–75 years (n = 3, 3%). One (1%) respondent preferred not to specify their age.

### Cat signalment

**Figure 2:**
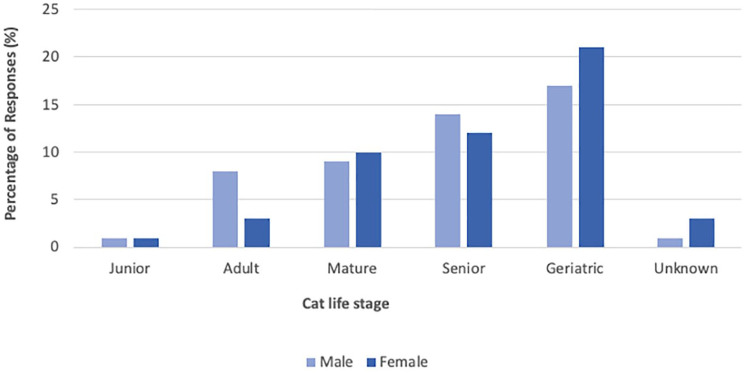
Sex and life stage of cats

In total, 47 (47%) cats were female neutered, two (2%) were female entire, 49 (49%) were male neutered and one (1%) cat was male entire. One (1%) owner was unsure of their female cat’s neuter status. Cat life stage and sex are presented in Figure 2. Of the cats, 55 (55%) were domestic shorthairs, 19 (19%) were domestic longhairs and 23 (23%) were pedigree; four (4%) owners did not know the breed of their cat.

### Owner-reported knowledge of their cat’s CKD

At the time of completing the survey, 10 (10%) owners reported that their cat was diagnosed with CKD less than 1 month prior, 20 (20%) between 1 and 6 months prior, 14 (14%) between 6 and 12 months prior, 24 (24%) between 1 and 2 years prior and 31 (31%) over 2 years prior; one (1%) owner was unsure when the diagnosis was made.

Of the owners, 73 (73%) had knowledge of their cat’s IRIS stage, of which 12 (16%) cats were reported to be in stage 1 CKD, 27 (37%) in stage 2, 23 (32%) in stage 3 and 11 (15%) in stage 4.

Concurrent disease was reported in 68 (68%) cats and more than one concurrent disease in 10 (10%) cats. The five most common concurrent diseases were systemic hypertension (n = 19, 30%), hyperthyroidism (n = 8, 12%), chronic enteropathy (n = 7, 10%), cardiomyopathy (n = 6, 9%) and neoplasia (n = 5, 7%).

Owners were asked to select the statement that best reflected their own understanding of their cat’s prognosis related to its CKD. Of the owners, 53 (53%) reported that they believed their cat had a non-favourable prognosis, with 32 (32%) owners selecting ‘poor’ prognosis and 21 (21%) selecting ‘guarded’ prognosis. In total, 17 (17%) owners reported they believed their cats had a ‘good’ prognosis and 29 (29%) a ‘fair’ prognosis. One (1%) owner reported that their understanding of their cat’s prognosis was unknown.

### Owner-reported CKD management

Of the owners, 76 (76%) fed their cat a prescription renal diet, with the majority (n = 44, 44%) feeding this diet exclusively. In total, 24 (24%) owners had never fed a prescription renal diet to their cat. Seventy (70%) owners stated a veterinary recommendation had influenced their decision to feed a renal prescription diet either solely or in conjunction with other influences (Table 1).

**Table 1:** Factors influencing decisions of owners of cats with CKD (n = 100) to feed a renal prescription diet

Factor	n (%)
Veterinarian recommendation	39 (39)
Veterinarian and internet recommendation (internet forums, group discussions or reading material online)	19 (19)
Veterinarian and veterinary nurse recommendation	5 (5)
Veterinarian and other recommendation	5 (5)
Internet recommendation (internet forums, group discussions or reading material online)	3 (3)
Other recommendation*	3 (3)
Veterinarian, internet and other recommendation	1 (1)
Internet and other recommendation	1 (1)
Veterinarian, internet and friend recommendation	1 (1)
No renal prescription diet fed	23 (23)

*Includes influences other than veterinarian, veterinary nurse, internet or friend recommendations Respondents could select all options that applied, and the response groups are therefore presented as composite groups based on those responses CKD = chronic kidney disease

In total, 69 (69%) owners reported that they were administering medications or supplements to their cat with CKD. Of the medications being administered, the most frequently reported were intestinal phosphate binders (n = 24, 35%), natural supplements (n = 18, 26%), antiproteinuric therapies (n = 17, 25%), amlodipine (n = 13, 19%) and appetite-stimulating drugs (n = 12, 17%) (Table 2).

**Table 2:** Medications and supplements being administered to cats with CKD

Medication or supplement	Frequency (n)	%
Phosphate binders		35
Calcium carbonate and chitosan (Ipakitine; Vetoquinol and ProNefra; Virbac)	23	
Aluminium hydroxide	1	
Natural supplements		26
Astro’s Oil Advanced Renal Care Formula	3	
Slippery elm	3	
CBD oil	2	
NutraRen (Nutravet)	2	
Kidney Support Gold (Pet Wellbeing)	2	
Astro’s Oil Nitrogen-Creatinine Scrub	1	
Astro’s Oil Renal Care Protein	1	
Salmon oil	1	
Green-lipped mussel	1	
Food-Grown Multi Strain Biotic (Wild Nutrition)	1	
Ubiquinol	1	
Antiproteinuric therapies		25
Benazepril	5	
Telmisartan	12	
Calcium channel blockers		19
Amlodipine	13	
Appetite stimulants		17
Mirtazapine	11	
Cyproheptadine (Periactin; Teva)	1	
Gastroprotectants		14
Famotidine	7	
Omeprazole	3	
Antiemetics		12
Maropitant	5	
Ondansetron	2	
Metoclopramide	1	
Laxatives/prokinetics		12
Lactulose	4	
Polyethylene glycol	3	
Cisapride	1	
Potassium supplements		9
Potassium gluconate, vitamins, amino acids and iron (Kaminox; VetPlus)	5	
Potassium gluconate (Tumil-K; Virbac)	1	
Antithyroid medications		9
Thiamazole	4	
Carbimazole	1	
Methimazole	1	
Analgesics		7
Amantadine	1	
Buprenorphine	1	
Meloxicam	1	
Robenacoxib	1	
Gabapentin	1	
Urinary tract supplements		6
Glucosamine, hyaluronic acid and l-tryptophan (Cystophan; Protexin Veterinary and Cystease; Ceva)	3	
D-mannose	1	
Antibiotics		4
Amoxycillin-clavulanate	3	
Joint supplements		4
Glucosamine and chondroitin (Seraquin; Boehringer Ingelheim)	2	
Gluosamine, green-lipped mussel and hyaluronic acid (YuMove; Lintbells)	1	
Liver supplement		1
*S*-adenosylmethionine and silybin (Denamarin; Protexin Veterinary)	1	
Probiotics		3
Probiotics, prebiotics, cobalamin, alpha-glucan butyrogenic and mucopolysaccharide (Pro-Kolin Enterogenic; Protexin Veterinary)	1	
Probiotics (Fortiflora; Purina)	1	
Other treatments		49
B vitamins (B12 and/or B3)	11	
Subcutaneous fluids	9	
Prednisolone	5	
Darbopoeitin	1	
Insulin	1	
Levothyroxine	1	
Timolol	1	
Ciclosporin	1	

Percentage was determined for each class of drug from the total number of owners reporting to be administering medications (n = 69) CKD = chronic kidney disease

The majority of owners (n = 28, 28%) presented their cat to a veterinary practice for monitoring related to CKD once or more every 1–2 months. A total of 13 (13%) owners presented their cat to the veterinary practice more than once a month, 24 (24%) once or more every 3–4 months, 16 (16%) once or more every 4–6 months, 15 (15%) once or more every 6–12 months, three (3%) once or more every 12–24 months and one (1%) less than once every 24 months.

### Owners’ experiences and feelings at diagnosis of CKD and at the time of survey completion

Owners were asked about their emotions and feelings at the time of their cat’s diagnosis of CKD as well as at the time of the survey. Table 3 presents the proportions of owners who experienced feelings of worry, anxiety or concern about different aspects of their cat’s CKD and the severity of these feelings. Extreme worry, anxiety or concern regarding their cat’s prognosis was reported by 72 (72%) owners at the time of diagnosis, although this decreased to 49 (50%) at the time of the survey. Extreme worry, anxiety or concern regarding their cat’s quality of life was reported by 63 (63%) owners, decreasing to 45 (46%) at the time of the survey. Over 60% of owners were either significantly or extremely concerned about going away on holiday or a work-related trip, both at diagnosis (n = 62, 62%) and at the time of survey completion (n = 63, 63%). Most survey respondents had some degree of worry, anxiety or concern for each of the statements, with the severity of the feelings only differing slightly between diagnosis and survey completion. There were some notable differences, which included a higher number of owners who reported not experiencing any anxieties or concerns towards changing their cat’s diet (n = 17, 17% vs n = 32, 33%) or administering medications (n = 20, 20% vs n = 33, 34%) at the time of survey completion compared with at the time of diagnosis of CKD.

**Table 3:** Reported frequency and severity of feelings of anxiety, concern or worry by owners about different aspects of their cat’s CKD care, at diagnosis (n = 100) and then at the time of taking the survey (n = 97)

Statement	Frequency of severity of feelings of anxiety, concern or worry					
		Not at all	Slightly	Moderately	Significantly	Extremely
Cost of treatment (medication, food and veterinary bills)	At diagnosis	23 (23)	17 (17)	30 (30)	13 (13)	17 (17)
	At survey completion	25 (25.8)	15 (15.5)	23 (23.7)	16 (16.5)	18 (18.6)
Prognosis of the disease	At diagnosis	1 (1)	4 (4)	8 (8)	15 (15)	72 (72)
	At survey completion	6 (6.2)	9 (9.3)	13 (13.4)	20 (20.6)	49 (50.5)
My cat’s quality of life	At diagnosis	3 (3)	4 (4)	6 (6)	24 (24)	63 (63)
	At survey completion	9 (9.3)	12 (12.4)	12 (12.4)	19 (19.6)	45 (46.4)
Giving medication to my cat	At diagnosis	20 (20)	14 (14)	27 (27)	20 (20)	19 (19)
	At survey completion	33 (34)	21 (21.6)	12 (12.4)	12 (12.4)	19 (19.6)
Leaving my cat at home	At diagnosis	23 (23)	20 (20)	12 (12)	18 (18)	27 (27)
	At survey completion	29 (29.9)	13 (13.4)	10 (10.3)	14 (14.4)	31 (32)
Letting my cat outside	At diagnosis	39 (39)	18 (18)	8 (8)	8 (8)	27 (27)
	At survey completion	38 (39.2)	13 (13.4)	12 (12.4)	5 (5.2)	29 (29.9)
Going on holiday or a work-related trip	At diagnosis	14 (14)	12 (12)	12 (12)	15 (15)	47 (47)
	At survey completion	18 (18.6)	9 (9.3)	7 (7.2)	15 (15.5)	48 (49.5)
Changing my cat’s food	At diagnosis	17 (17)	17 (17)	21 (21)	23 (23)	22 (22)
	At survey completion	32 (33)	14 (14.4)	14 (14.4)	20 (20.6)	17 (17.5)
Maintaining my cat’s water intake	At diagnosis	21 (21)	13 (13)	21 (21)	25 (25)	20 (20)
	At survey completion	29 (29.9)	14 (14.4)	15 (15.5)	17 (17.5)	22 (22.7)
Regular veterinary visits	At diagnosis	26 (26)	15 (15)	20 (20)	19 (19)	20 (20)
	At survey completion	28 (28.9)	16 (16.5)	15 (15.5)	16 (16.5)	22 (22.7)
My relationship with my cat	At diagnosis	48 (48)	12 (12)	13 (13)	11 (11)	16 (16)
	At survey completion	50 (51.5)	13 (13.4)	11 (11.3)	10 (10.3)	13 (13.4)

Data are n (%) CKD = chronic kidney disease

A total of 41 free-text responses described owners’ additional worries, anxieties or concerns with regard to their cat’s CKD. Of the 41 free-text responses, 35 could be categorised into subjects in which more than one owner had concerns. Subjects identified with exemplar quotes included the following:

- concerns relating to diet (n = 6): ‘We are at the very early stages of our cat being diagnosed with CKD. It is extremely worrying, we love our cat very much and want the best care possible for him. Right now we are unsure what diet he should be eating. He has tried the renal food but won’t eat it. We tried him on several higher quality protein foods but he’s not very keen on them either. I wish there was more information available’;
- veterinarian’s depth of knowledge (n = 8): ‘The vet did not seem to be very clued up on CKD’;
- veterinarian’s willingness to consider alternative treatment options presented by the owner (n = 3): ‘Getting vet to consider other options of treatment’;
- handling of the cat at a veterinary practice (n = 4): ‘My cat is a terror at the vet’s and has to be muzzled and sedated before she can be examined, so I don’t take her regularly as the sedation damages her kidneys’;
- cat’s behaviour (n = 5): ‘She hated
- having the medication squirted into her mouth (she wouldn’t eat it mixed into her food) so it made me feel very upset and stressed that I had to put her through it at the end of her life’;
- effect of CKD diagnosis on other cats in the household (n = 3): ‘The effect on my other two cats regarding feeding diet or if we lost him as they are his mum and sister and very close’; and owner’s ability to recognise pain or clinical signs of CKD (n = 6): ‘Worried about if she was in pain and not knowing when she was’.

**Figure 3:**
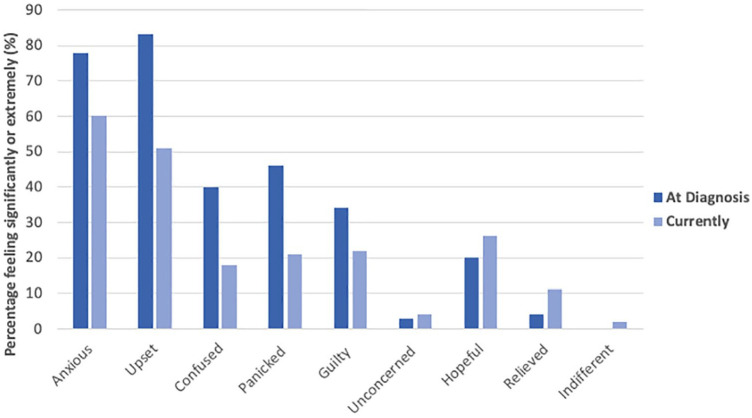
Percentage of owners who reported experiencing various emotions with extreme or significant severity at diagnosis of chronic kidney disease and at the time of survey completion

Figure 3 presents owners’ emotions ranked at the time when their cat was diagnosed with CKD and at the time of survey completion. At the time of diagnosis, most owners felt either extremely or significantly upset (n = 83, 83%), anxious (n = 78, 78%), panicked (n = 46, 46%) and/or confused (n = 40, 40%). At the time of survey completion, decreased feelings of confusion (n = 18, 18.6%) and panic (n = 21, 21.6%) were reported, and an increased number of owners (n = 20, 20.6%) ranked upset as ‘not at all’. Anxiety remained a highly ranked emotion, with 88 (90.7%) owners still experiencing some degree of anxiety surrounding their cat’s care compared with 99 (99%) experiencing some degree of anxiety at the time of diagnosis.

The agreed and strongly agreed categories describing owners’ experiences of caring for a cat with CKD (Table 4) were combined. In total, 66 (66%) owners agreed that their daily routine had changed a lot since their cat was diagnosed with CKD, 37 (37%) owners agreed that caring for their cat negatively impacted or restricted their own life, and 70 (70%) owners agreed that they felt supported by their veterinary practice with regard to their cat’s care. When it came to making decisions about their cat’s care, 80 (80%) owners agreed that they felt comfortable making decisions with a veterinary surgeon compared with 48 (48%) feeling comfortable discussing with a veterinary nurse. Of the owners, 78 (78%) agreed that they felt knowledgeable about feline CKD, 80 (80%) agreed that they were able to assess their cat’s quality of life and 65 (65%) agreed that they felt confident in being able to monitor the progression of their cat’s CKD. Over half of respondents (n = 52, 52%) agreed that their cat’s condition had improved since beginning treatment and 49 (49%) agreed that being a caregiver to their cat has given them purpose and a sense of accomplishment.

**Table 4:** Owners’ agreement with statements relating to experiences of caring for their cats with CKD (n = 100)

Statement	Strongly agree	Agree	Neither agree nor disagree	Disagree	Strongly disagree
My daily routine has changed a lot since my cat was diagnosed with CKD	31 (31)	35 (35)	14 (14)	13 (13)	7 (7)
The care of my cat has negatively impacted/restricted my life	14 (14)	23 (23)	20 (20)	18 (18)	25 (25)
I feel supported by my veterinary practice with regard to my cat’s care	42 (42)	28 (28)	20 (20)	7 (7)	3 (3)
I feel comfortable making decisions with a veterinarian about my cat’s care	39 (39)	41 (41)	15 (15)	5 (5)	0 (0)
I feel comfortable making decisions with a veterinary nurse about my cat’s care	26 (26)	22 (22)	38 (38)	12 (12)	2 (2)
I feel I am knowledgeable about CKD in cats	28 (28)	50 (50)	12 (12)	6 (6)	4 (4)
I am able to determine/assess my cat’s quality of life	26 (26)	54 (54)	12 (12)	7 (7)	1 (1)
I feel confident I am able to monitor the progression of my cat’s CKD	28 (28)	37 (37)	25 (25)	7 (7)	3 (3)
I feel my cat’s condition has improved since starting treatment	22 (22)	30 (30)	30 (30)	10 (10)	8 (8)
Being a caregiver to my cat has given me purpose and a sense of accomplishment	26 (26)	23 (23)	33 (33)	11 (11)	7 (7)

Data are n (%)

CKD = chronic kidney disease

A total of 33 (33.6%) owners reported that they felt their relationship with their cat had improved since the diagnosis of CKD (17.3% reported it had improved a lot and 16.3% reported it had improved a little). Fifty-four (55.1%) owners reported their relationship with their cat had stayed the same, seven (7.1%) reported that it had worsened and four (4.1%) were unsure.

## Discussion

This is the first study reporting UK-based owner experiences of caring for a cat with CKD. Although knowledge of their cat’s CKD and appropriate management was generally in line with expert guidelines,^2^ the finding that many owners reported experiencing feelings of anxiety associated with caring for their cat was of concern. This highlights the impact that veterinary professionals including veterinary nurses may have in providing greater support.

A high percentage of owners (73%) in the present study had knowledge of their cat’s IRIS stage, suggesting that clinicians are conducting staging and informing owners accordingly. This finding contrasts with a large cohort study that reported only 19.8% of UK cats diagnosed with CKD had IRIS stages recorded in their clinical notes.^11^ Respondents in the present study were likely to have been highly committed, with some knowledge of feline CKD, influencing the results. IRIS staging was based solely on owner recollection and therefore the reliability of the data is uncertain.

More than three-quarters of owners reported that they were feeding a renal diet, with 44% feeding it exclusively. Veterinary recommendation was reported to influence 70% of owners in their decision to feed a renal diet, highlighting the important role that veterinarians play in this key aspect of feline CKD management. This finding is in line with a previous study reporting increased compliance in feeding a renal diet upon veterinary recommendation.^5^ Despite this, a proportion of free-text answers were related to owner concerns about diet, most notably a lack of understanding regarding appropriate diets and concerns about their cat’s tolerance of new diets. These findings suggest that although a veterinary recommendation was highly influential in decisions to feed a renal diet, not all owners felt adequately supported or informed regarding transitioning to a renal diet.

Of cats receiving medications in the present study, the most frequently administered was a phosphate binder (n = 24, 35%). Many owners (n = 18, 26%) were administering natural supplements to their cat. It is unclear if these were recommended by veterinary professionals or the result of owners’ independent decisions. Natural supplements are unregulated, often lack evidence base and, in humans, it is recognised that many have potentially harmful ingredients that should be avoided in patients with CKD.^12,13^ At least one of the supplements reported in the present study contained a herb (astragalus) that the National Kidney Foundation recognises as harmful to patients with CKD (www.kidney.org/atoz/content/herbalsupp) and it was not possible to ascertain the ingredients of all the supplements. Some owners had expressed concerns about their veterinarian’s willingness to consider alternative treatment options, suggesting a potential barrier to conversation regarding the use of supplements in cats with CKD.

Almost all owners in the present study (99%) reported experiencing some degree of anxiety at the time of receiving their cat’s CKD diagnosis, with extreme anxiety described by 58% of owners. An owner’s anxiety may be heightened when receiving bad news, and the intensity depends on different factors, including situational and personal.^14,15^ Many owners (68%) reported that their cat had concurrent disease and this may also contribute to feelings of anxiety. The leading causes of anxiety at CKD diagnosis were related to disease prognosis and quality of life. Of the owners, 53 (53%) reported that they believed their cat had a non-favourable prognosis. Many cats with stable early-stage disease will survive with a good quality of life for a significant period of time. Indeed, one study reported that cats with tubulointerstitial disease consistent with CKD survived longer than cats that did not.^16^ Veterinary professionals can help to alleviate anxiety by educating owners regarding prognosis and quality-of-life monitoring. Ensuring owners are involved with ongoing monitoring for disease progression and management plans can elicit a sense of empowerment and help decrease anxiety.^17^

At the time of survey completion, 90.7% of owners still reported some degree of anxiety related to their cat’s care. This finding suggests that despite owners reporting feeling more informed about CKD in cats, 52% feeling that their cat’s condition had improved and 49% feeling a sense of purpose when caring for their cat, these improvements did not contribute to a significant reduction in anxiety. Caregiver burden is linked to anxiety and other symptoms of depression.^7^ Factors contributing to caregiver burden in veterinary clients involve changes in routine and limitations to work or social life.^7^ These factors were noted within survey responses, with 63% of owners reporting to be anxious about going away and 66% agreeing that their daily routine had changed a lot since their cat’s CKD diagnosis. A consideration relevant to these findings is that the survey was distributed within social media feline CKD support groups and owners choosing to join these may have done so to seek support relating to the negative emotions they were experiencing. Furthermore, 94% of the population consisted of women, in whom there is a higher prevalence of anxiety disorders than men.^18^

It has been suggested that there are different aspects contributing to owners’ negative emotions; these are anticipatory grief, caregiver burden and owner’s quality of life.^19^ It has also been suggested that these do not directly translate to one another and so should be evaluated separately.^19^ The findings of the present study suggest that it is highly probable that owners of cats with CKD experience each of these. Further studies should evaluate these individually in owners of cats with CKD using validated assessments such as the caregiver grief scale,^19,20^ caregiver burden using the Zarit Burden Interview,^19,21^ and quality of life enjoyment and satisfaction questionnaire.^19,22^

Although this study has highlighted negative emotions that can suggest caregiver burden, there are also some positive findings. One-third of owners felt that the diagnosis of CKD had improved their relationship with their cat, half reported feeling a sense of purpose in caring for their cat with CKD and 70% agreed that they felt supported by their veterinary practice (with 81% presenting their cats for monitoring within 6 months of the previous visit).

In humans, there are significant benefits to nurse involvement in the care and support of patients with CKD, including improved renal outcomes,^23^ enhanced adherence to phosphate binders^24^ and patient satisfaction with quality of life.^25^ Opportunities identified in the present study in which veterinary nurses could provide additional support to owners include medicating cats, transitioning to a renal diet and quality-of-life monitoring. However, only 48% of the owners in the present study felt comfortable discussing their cat’s care with a veterinary nurse. The reasons for this were unclear. It is possible that the owners in the study did not understand the role of veterinary nurses^26^ or that they may not have experienced a consultation with a veterinary nurse. Future studies should address owners’ perceptions of the veterinary nurse’s role within feline CKD management and evaluate the benefits of nurse-led renal clinics. However, it is recognised that there can be obstacles to these clinics, such as finances and lack of knowledgeable nurses.^27^

There are several limitations to the study. The study was only open to UK participants and therefore its findings may not directly translate to the attitudes of cat owners from other countries. The study included data from 100 respondents, which may be considered a relatively small sample size. There is also the potential for bias in the study population, with respondents likely representing a motivated and committed group of owners, possibly with good knowledge of CKD in cats. Diagnostic criteria to define CKD and access to clinical records were not available to confirm CKD and the diagnosis was owner-reported. Some questions in the survey required owners to retrospectively report past events and emotions, which may have led to recall bias. Finally, as with all survey studies, social desirability bias may also have influenced responses.

## Conclusions

The International Society of Feline Medicine consensus guidelines for the management of feline CKD state that ‘a good relationship and good communication between the clinic and cat’s owner is vital’ for successful management of CKD.^2^ This study has highlighted the anxiety experienced by the majority of owners caring for a cat with CKD, which may suggest caregiver burden. It is important to draw attention to this finding so that the veterinary team can adopt empathetic communication. We have identified opportunities in which the veterinary team, including nurses, can provide greater education and support to enhance the relationship and communication with owners of cats with CKD, helping to alleviate anxiety and ensure ongoing successful CKD management.

## Supplemental Material

Supplemental Material Copy of the survey used for this study, as it appeared on Bristol Online Surveys.
