# Supplementary material for: Owners’ experiences of caring for cats with chronic kidney disease in the UK
Source: J Feline Med Surg. 2025 Apr 16;27(4):1098612X251314769. doi: 10.1177/1098612X251314769 (PMC12035312; doi:10.1177/1098612X251314769)
Supplement: Supplemental Material [file sj-docx-1-jfm-10.1177_1098612X251314769.docx]

Owner Experiences in Caring for Cats with Chronic Kidney Disease in the UK.

Section A : Owner Information

1: What is your gender? Please select exactly 1 answer(s) Multiple Choice closed

- Male
- Female
- Other
- Prefer not to say

2: What is your age? Please select exactly 1 answer(s) multiple choice closed

- Under 18
- 18-25
- 26-35
- 36-45
- 46-55
- 56-65
- 66-75
- Over 75
- Prefer not to say

3: Do you live within the United Kingdom? Please select exactly 1 answer(s) multiple choice closed

- Yes
- No

**Section B: Cat information**

4: Is your cat male or female? Please select exactly 1 answer(s) multiple choice closed

- Male
- Female
- Unknown

5: Is your cat neutered or not neutered? Please select exactly 1 answer(s) multiple choice closed

- Neutered
- Not Neutered
- Unknown

6: What is your cat’s approximate age? Please select exactly 1 answer(s) multiple choice closed

- Kitten (Birth- 6months)
- Junior (7 months- 2 years)
- Adult (3-6 years)
- Mature (7-10 years)
- Senior (11-14 years)
- Geriatric (15+ years)

7: What breed is your cat? Please select exactly 1 answer(s) multiple choice closed

- Domestic Short Hair
- Domestic Long Hair
- Pedigree (please state breed)
- Unknown

8: Is your cat an Indoor/house cat or outdoor cat? Please select exactly 1 answer(s) multiple choice closed

- Indoor Cat (Spends >90% of their time indoors)
- Outdoor Cat (Spends >90% of their time outdoors
- Indoor/Outdoor Cat
- Unknown

Section C: CKD

9: How many people other than yourself are regularly involved in your cat’s care? Please select exactly 1 answer(s) multiple choice closed

- 1
- 2
- 3
- 4+
- No other people

10: Approximately how long ago was your cat diagnosed with Chronic Kidney Disease? Please select exactly 1 answer(s) multiple choice closed

- Less than 1 month
- Less than 6 months
- Less than 12 months
- Less than 2 years
- Over 2 years
- Unknown

11: Has your cat ever had to be admitted into a veterinary hospital overnight or longer period for reasons relating to their kidney disease? Please select exactly 1 answer(s) multiple choice closed

- Yes, once
- Yes, more than once
- No, never
- Unknown

12: Below is a list of signs that may be seen in cats with Chronic Kidney disease, for each sign please rank the severity in your cat. **1= Not seen , 2= Not very severe, 3 = Quite severe, 4= Severe 5= Very severe.**

Likert scale question

Please don't select more than 1 answer(s) per row. Please select exactly 10 answer(s)

- Reduced Appetite
- Weight Loss
- Increased Thirst
- Increased Urination/ passing larger volumes of urine
- Lethargy
- Vomiting
- Diarrhoea
- Poor Coat Quality
- Bad Breath
- Muscle Weakness/ Wastage

13: Has your cat been diagnosed with any of the following concurrent diseases? Select all that apply

multiple choice closed

- Diabetes
- Hyperthyroidism
- Cancer
- Irritable Bowel Disease
- Heart Disease
- High Blood Pressure (Hypertension)
- Other please specify
- No other diseases

14: Do you know what stage of kidney disease your cat is in? If yes please indicate below using the International Renal Interest Society (IRIS) CKD staging. Please select exactly 1 answer(s) multiple choice closed

- IRIS Stage 1
- IRIS Stage 2
- IRIS Stage 3
- IRIS Stage 4
- Unknown

15: What is your understanding of your cat’s prognosis? Please select from the descriptions below***.*** Please select exactly 1 answer(s) multiple choice closed

- Good – Favourable outcome is expected and CKD may be easily managed
- Fair – Favourable outcome possible and CKD is manageable
- Guarded – Possible outcome is unknown
- Poor – Non-favourable outcome is expected
- Unknown.

Section D: Diet and Medication/Supplements

16: Do you feed your cat a prescription renal/kidney diet? Please select exactly 1 answer(s) multiple choice closed

- Yes, all the time
- Yes, sometimes
- No
- Unknown

17: What influenced your decision to feed your cat a renal prescription diet? Select all that apply

Multiple choice closed + free-text other option

- Vet recommendation
- Vet Nurse recommendation
- Pet Shop recommendation
- Internet recommendation (internet forums, group discussions or reading material online)
- Friend recommendation
- Breeder recommendation
- Other- specify
- I don’t feed a renal prescription diet

18: How would you describe your cat’s appetite? Please select exactly 1 answer(s) multiple choice closed

- My cat has no loss of appetite
- My cat occasionally loses appetite but can be easily encouraged to eat during these periods
- My cat occasionally loses appetite and it is difficult to encourage eating during these periods
- My cat regularly loses appetite but can be easily encouraged to eat during these periods
- My cat regularly loses appetite and it is difficult to encourage eating during these periods

19: Do you give your cat any medication and/or supplements? Please select exactly 1 answer(s)

multiple choice closed

- Yes
- No
- Unknown

20: Do you know the name of the medication and/or supplements? Please select exactly 1 answer(s)

Multiple choice closed + free-text specify option

- Yes, please specify
- No
- My cat does not receive any medication and/or supplement

21: How often do you give the medications and/or supplements to your cat? Please select exactly 1 answer(s) multiple choice closed

- Once a day
- 2 times a day
- 3 times a day
- 4+ times a day
- Other please specify
- My cat does not receive any medication and/or supplement

22: If you administer any medication to your cat how well does your cat tolerate receiving the medication/supplements? Please select exactly 1 answer(s) multiple choice closed

- Does not tolerate
- Tolerates some of the time
- Tolerates most of the time
- Tolerates all of the time

23: Please rank how easy or difficult you find it to administer the following types of medications to your cat. **1= very easy 2= easy, 3 = neither easy or difficult, 4= difficult, 5= very difficult**

Likert scale question

Please don't select more than 1 answer(s) per row. Please select exactly 4 answer(s).

- Liquid
- Tablet
- Powder
- Injection

23b: If you give any other type of medication not listed above, please specify below:

open free-text sub question

- Free text to answer

24: Do you assess your cat’s hydration? Please select exactly 1 answer(s)

multiple choice closed

- Yes, all the time
- Yes, sometimes
- No
- Unknown

25: If you assess your cat’s hydration how do you do this?

Open free text

- Free text to answer

26: Do you utilise any of the following to provide additional fluid intake? Select all that apply

multiple choice closed + free-text other option

- Feed a wet diet
- Use multiple different water bowels (plastic, ceramic, metal and glass)
- Use a cat water fountain
- Offer flavoured water e.g. chicken/tuna/prawn
- Using different sources of water e.g. tap water, rain water or mineral water
- Add further water to food
- Intermittent fluid therapy at vets
- Intermittent subcutaneous fluid therapy at vets or home.
- Other
- None of the above

Section E : Living with your CKD Cat

27: How often do you take your cat to the vet for check-ups? Please select exactly 1 answer(s)

multiple choice closed

- Once or greater a month
- Once or greater every 1-2 Months
- Once or greater every 3-4 Months
- Once or greater every 4-6 Months
- Once or greater every 6-12 Months
- Once or greater every 12-24 Months
- Less than once every 24 Months
- Never

28: When your cat was FIRST diagnosed please rank how anxious, worried or concerned you felt towards the following with regards to your cat’s chronic kidney disease and care? **1= not at all, 2= slightly, 3= moderately, 4=significantly, 5=extremely**

Likert scale question

Please don't select more than 1 answer(s) per row. Please select exactly 11 answer(s).

- Cost of treatment (medication, food and vet bills)
- Prognosis of the disease
- My cat’s quality of life
- Giving medication to my cat
- Leaving my cat at home
- Letting my cat outside
- Going on holiday or a work-related trip
- Changing my cat’s food
- Maintaining my cat’s water intake
- Regular vet visits
- My Relationship with my cat

28b: If you had any other specific concerns not listed above please elaborate in the box below;

Open free-text sub-question

- Free text to explain.

29: Please rank how anxious, worried or concerned you CURRENTLY feel towards the following with regards to your cat’s chronic kidney disease and care? **1= not at all, 2= slightly, 3= moderately, 4=significantly, 5=extremely**

Likert scale question

Please don't select more than 1 answer(s) per row. Please select exactly 11 answer(s).

- Cost of treatment (medication, food and vet bills)
- Prognosis of the disease
- My cat’s quality of life
- Giving medication to my cat
- Leaving my cat at home
- Letting my cat outside
- Going on holiday or a work-related trip
- Changing my cat’s food
- Maintaining my cat’s water intake
- Regular vet visits
- My Relationship with my cat

29b: If you currently have any other specific concerns not listed above please elaborate in the box below;

Free-text sub-question

- Free text to explain.

30: From the list of possible emotions below please rank how you felt when your cat was FIRST diagnosed with chronic kidney disease? **1= not at all, 2= slightly, 3= moderately, 4=significantly, 5=extremely**

Likert scale question

Please don't select more than 1 answer(s) per row. Please select exactly 9 answer(s).

- Anxious
- Upset
- Confused
- Unconcerned
- Panicked
- Guilty
- Hopeful
- Relieved
- Indifferent

30b: If you felt any other specific emotion please specify in box below: #

Free text sub-question

- Free text to explain.

31: From the list of possible feelings please rank how you CURRENTLY feel about your cat’s chronic kidney disease? **1= not at all, 2= slightly, 3= moderately, 4=significantly, 5=extremely**

Likert scale question

Please don't select more than 1 answer(s) per row. Please select exactly 9 answer(s).

- Anxious
- Upset
- Confused
- Unconcerned
- Panicked
- Guilty
- Hopeful
- Relieved
- Indifferent

31b: If you currently feel any other specific emotion please specify in box below:

Free text sub-question

- Free text to explain

32: Do you feel your relationship with your cat has improved since being diagnosed? Please select exactly 1 answer(s)

multiple choice closed

- Yes, a lot
- Yes, a little
- It is the same
- No, it has got worse
- Unsure

33: How do you source information about chronic kidney disease in cats? select all that apply

multiple choice closed + free-text other option

- From a vet
- From a vet nurse
- Internet forums/groups
- Internet search
- From books
- From brochures
- From other owners of cats with chronic kidney disease
- Other please specify
- I do not look for information

| Strongly Agree | Agree | Neither agree nor disagree | Disagree | Strongly Disagree |
| --- | --- | --- | --- | --- |

34: Below is a range of statements relating to experiences of caring for your CKD cat, please select from the option if you agree or disagree. Please don't select more than 1 answer(s) per row. Please select exactly 10 answer(s).

Likert scale question

- My daily routine has changed a lot since my cat was diagnosed with CKD.
- I am able to determine/assess my cat’s quality of life
- I feel my cat’s condition has improved since starting treatment
- I feel comfortable making decisions with my vet about my cat’s care
- I feel comfortable making decisions with a vet nurse about my cat’s care
- I feel I am knowledgeable on CKD in cats
- The care of my cat has negatively impacted/ restricted my life
- I feel confident that I am able to monitor the progression of my cat’s CKD
- I feel supported by my veterinary practice with regards to my cat’s care
- Being a care-giver to my cat has given me purpose and a sense of accomplishment

35: If you have any additional thoughts or comments regarding your experience of caring for your cat with CKD that have not been covered by this survey please include here: open/free-text question

- Free text to answer
